# Utilization of animal by-products as sources of bioactive compounds and FBS alternatives for cultured meat: a comprehensive review

**DOI:** 10.1007/s44463-025-00035-8

**Published:** 2026-02-11

**Authors:** Juhyun Lee, Da-Young Lee, Ermie Jr. Mariano, Ji Won Park, Seok Namkung, So Young Choi, Woo Jin Lee, Ye Won Shin, Chae Hyeon Bok, Colin Venter, Younsu Lee, Sun Jin Hur

**Affiliations:** 1 Department of Animal Science and Technology, Chung-Ang University, Anseong, Republic of Korea; 2 Department of Pet Health, Kwangju Women’s University, 40 Gwangjuyeodai-gil, Gwangsan-gu, Gwangju, 62396 Republic of Korea

**Keywords:** Slaughterhouse by-products, Animal blood, Fetal bovine serum, Cultured meat, Waste valorization, Cell culture media

## Abstract

Slaughterhouse by-products have been widely utilized in food, pharmaceutical, and biomaterial applications across Asia, Europe, and the Americas. This review examines current research on enhancing the value and utilization of these by-products, with a particular focus on their potential as alternatives to fetal bovine serum (FBS), a critical component of cell culture media. Among these, livestock and poultry blood—produced at over 30 million tons annually—is of particular interest; however, only approximately 30% is currently used in food products or additives. Recent studies suggest that serum derived from bovine, porcine, and chicken blood may serve as viable substitutes for FBS. Recent studies have suggested that serum derived from adult bovine, porcine, and chicken blood may partially substitute for FBS, with replacement efficiencies reported to reach up to approximately 80%, depending on the species. Inedible by-products, including feathers, bones, leather, and hooves, are primarily processed into fertilizers and animal feed due to their high protein and mineral content. Despite the considerable volume of slaughter by-products generated, their application in cell culture systems and other high-value industries remains underdeveloped. Promoting the industrial utilization of these materials could not only improve resource efficiency and reduce environmental impact but also contribute to the sustainability and economic value of the livestock sector.

**Supplementary Information:**

The online version contains supplementary material available at 10.1007/s44463-025-00035-8.

## Introduction

The slaughtering of livestock inevitably generates a wide range of by-products, yet their utilization remains limited, leading to increased disposal costs, environmental pollution, and reduced economic value (Jeon, 2013; Alao et al., 2017). Although such by-products are employed in various fields—particularly as food ingredients—their application rate is still disproportionately low compared to the total volume produced (Ahn et al., 2019; Limeneh et al., 2022). Therefore, enhancing the efficient use of these materials remains an ongoing challenge for the livestock industry. In response, increasing research efforts have focused on converting slaughterhouse by-products into valuable resources for food, pharmaceuticals, and animal feed sectors.

Simultaneously, the commercialization of cultured meat faces two major technical challenges: lowering cultivation costs an optimizing cell culture performance (Lee & Hur, 2025). Serum supplements, which are indispensable for most cell culture systems, constitute the largest cost factor while being critical for maintaining cellular stability and efficiency. Therefore, replacing costly fetal bovine serum (FBS) with effective and economically feasible alternatives is one of the most urgent challenges, as it is critical not only for cost reduction but also for sustaining culture performance. FBS, which is collected from the heart of bovine fetuses during the slaughter of pregnant cows, has long been a controversial and ethically debated component in cell culture (Van der Valk et al., 2018). With the rapid growth of the cultured meat industry, demand for FBS has risen sharply, amplifying both ethical and economic concerns.

Animal blood collected from slaughterhouses is rich in nutrients and bioactive compounds, including proteins and iron, offering significant potential not only as a high-value food ingredient but also as a component of cell culture media for cultured meat production (Lynch et al., 2017; Lee et al., 2024b). According to the Cheng et al. (2024), over 30 million tons of livestock and poultry blood are produced globally each year, yet only about 30% is utilized for food or additive purposes. The remainder is primarily used for pet food and fertilizer, or discarded altogether. In contrast to fetal bovine serum (FBS), which requires the additional sacrifice of the fetus for research purposes, the use of slaughterhouse blood represents the valorization of an unavoidable by-product of conventional meat production that is otherwise destined for disposal. While the cultured meat industry aims to minimize reliance on animal-derived materials, the ethical implications of using slaughterhouse blood should be weighed against its long-standing role in the livestock sector and its potential to reduce waste and environmental impact.

In addition to blood, a wide range of internal and external organs—such as the head, liver, kidneys, heart, small intestine, large intestine, and skin—are also obtained during slaughter and present significant potential for applications in cell culture systems, as well as in food and pharmaceutical industries (Jayathilakan et al., 2012; Ahn et al., 2019). However, research into their utilization in high-value sectors remains limited. Given the pressing need to improve sustainability and resource efficiency in livestock production, exploring the valorization of these by-products is both timely and necessary. Therefore, this review aims to examine the potential of slaughterhouse by-products—particularly animal blood—not only as sustainable, cost-effective alternatives to FBS in cultured meat production, but also as a foundation for expanding the scope of by-product utilization into cell-based systems. By highlighting emerging applications and current knowledge gaps, this work seeks to support the development of more efficient, ethical, and environmentally responsible approaches to by-product utilization.

## Definition and use of slaughterhouse by-products

Slaughterhouse by-products are secondary materials generated during the meat processing process, including blood, bones, meat scraps, leather, and adipose tissue (Demaman et al., 2025). These by-products are broadly categorized as edible and inedible (Ahn et al., 2019; Limeneh et al., 2022; Alibekov et al., 2024). Edible by-products include organs such as the heart, liver, and kidneys, while inedible by-products encompass materials such as hooves and bristles (Ahn et al., 2019; Al-Zohairi et al., 2023). Edible by-products are further categorized into primary by-products—such as blood, heads, and viscera obtained during slaughter—and secondary by-products—such as bones, fat, and trimmings produced during further processing. These materials are utilized in various sectors of the food industry (Ahn et al., 2019). Blood is typically collected during exsanguination, while viscera are collected during carcass splitting.

In terms of yield, slaughterhouse by-products account for a significant portion of live weight. On average, by-products constitute approximately 55% of live weight, with cattle accounting for about 40% and pigs about 30%. (NIAS, 2020; NIAS, 2021; Woodgate, 2023; Alibekov et al., 2024). Despite this volume, a significant portion of by-products remains underutilized and is often discarded. As global livestock production continues to rise, the amount of by-products generated is also increasing (Limeneh et al., 2022). However, the utilization rate remains low, contributing to resource inefficiency and environmental issues. Key contributing factors include fluctuations in livestock numbers, inconsistent by-product supply, high disposal costs, unpleasant odors, and the generation of wastewater sludge—all of which exacerbate environmental pollution (Rural Development Administration, 2012).

In Korea, the trend mirrors the global situation. Although by-product generation has increased alongside meat production, their overall market value has declined. Moreover, shifting consumer preferences toward specific parts have decreased the demand for others, increasing reliance on imports for certain by-products. Annually, Korea produces approximately 50,000 tons of cattle blood and 10,000 tons of pig blood; yet only 60–70% is utilized, with the rest being discarded (Choi, 2013). In particular, pig by-products face unique challenges, including unsanitary processing practices and inefficient distribution systems, often resulting in persistent odors due to improper handling (Jeon, 2013; Song et al., 2015). These issues—combined with changing consumption patterns, hygiene-conscious consumer trends, and weak production and distribution infrastructures—contribute to the large-scale disposal of domestic slaughter by-products.

In response, numerous studies have focused on the high-value utilization of these materials. Slaughter by-typically contain 15–22% protein (Kang et al., 2014; Limeneh et al., 2022), making them valuable for extracting bioactive peptides used in functional food applications (Ryder et al., 2016; Toldrá et al., 2016). Inedible by-products, such as feathers, bones, hide, and hooves, are also rich in protein and minerals, and are commonly repurposed as raw materials for fertilizers and animal feed. Increasing attention is now being directed toward their potential as sustainable environmental resources (Limeneh et al., 2022; Irshad & Sharma, 2015).

### Visceral by-products

Visceral by-products include the liver, heart, lungs, stomach, pancreas, spleen, kidneys, and, in the case of poultry, gizzards (Umaraw et al., 2025). Edible visceral by-products require processing, including bloodletting, collection, cleaning, preparation, refrigeration, packaging, and cooling (Jayathilakan et al., 2012). A global overview of edible by-products and their characteristics is presented in Table 1.

**Table 1:** Foods materials using animal slaughter by-products

Animals	By-products	Products	Characteristics	Country	Reference
Cattle	Bone	Beef bone broth protein powder	• Derived from dehydrated beef bone broth • High in protein and nutrients • Low in carbohydrates, suitable for ketogenic diets	US	Bulk Supplements, 2024
		Beef bone broth	• Prepared by simmering bovine bones and joints • Rich in protein and collagen for bone and skin health • Convenient for adding umami flavor to soups or pasta dishes	UK	Freja Foods, 2025
	Heart	Head cheese	• Traditional European offal-based sausage • Meat jelly prepared from parts of the animal’s head • Preparation methods vary by region	US	US Wellness Meats, 2022
		Beef & organ meat sticks	• Prepared from beef, heart, and liver in collagen casings • Air-dried, free from chemical additives • Convenient, health-conscious snack option	US	Lineage Provisions, 2025
	Liver	Chicken nuggets	• Addition of bovine liver to chicken nuggets • Increased moisture, ash, and fat contents • Improved antioxidant activity	Pakistan	Mehmood et al., 2024
		Carnivore crumbs liver	• Crispy snack made from bovine liver • High in nutritional value and protein content • No added sugars, suitable for ketogenic diets	US	Carnivore Crisps, 2025
	Stomach	*Menudo*	• Traditional Mexican soup with beef broth and stomach • Seasoned with spices • Convenient canned packaging for storage and consumption	US	Juanita’s Foods, 2024
	Tongue	Cured ox tongue	• Suitable as a topping for sandwiches or salads • Canned for extended shelf life and easy storage	UK	Marks and Spencer, 2025
		Jellied calves tongue	• Brined calf tongue processed with gelatin broth • Encased in fibrous casing for setting • Ready-to-slice and serve	US	Paulina Market, 2025
Pig	Blood	Sundae	• Korean dish using stuffed livestock intestines • Filled with seasoned meat, vegetables, and porcine blood • Steamed or boiled, utilizes animal by-products like blood and organs	South Korea	Sohn et al., 1999
		Black pudding	• Sausage made primarily from pig’s blood, popular in Europe • Rich in essential micronutrients: vitamins, iron, and zinc • High in blood-derived proteins and lysine	EU	Anjos et al., 2019
	Bone	Pork bone broth	• Prepared by roasting pig bones and simmering with water and vinegar for 24 h • Produces nutrient-rich broth • Commonly used as a soup base	US	Mama Tong Soup, 2025
	Liver	Hamburger patties	• Patties with 5% porcine liver • Enhanced sensory quality • Cost-effective use of liver by-products • Incorporates functional liver components	South Korea	Choi et al., 2017
		Pork liver *pâté*	• Soft, spreadable liver paste • Nutritious and low-calorie • Used as a snack or sandwich filling	EU	Essentia Protein Solution, 2025
	Skin	*Chicharrónes*	• Pork skin contains 0% carbohydrates per 14 g serving • Commercially marketed as a high-protein snack • Suitable for low-carb diets	Spain	4505 meats, 2025
		Kaeb moo	• High-protein pork skin snack • 0% carbs per 14 g serving	Southeast Asia	Sriwattana et al., 2012
Chicken	Feet	Charcoal-grilled boneless chicken feet	• Deboned and steamed • Deep charcoal flavor infused through grilling	Korea	Foodmax, 2020
		Frozen chicken feet	• Raw, chilled chicken feet • Convenient for various recipes	France	Edenfarmer, 2023
	Gizzard	Generic chicken gizzards	• 100% natural, no additives • Rich source of lean protein • Used in gumbo and stews, or as a crispy snack	US	Sanderson Farms, 2025
		Fried chicken gizzard	• Crispy exterior and chewy interior • Popular as a flavorful snack	Korea	Lifedryfish, 2024
	Heart	Grilled salted skewer	• Seasoned chicken sold on skewers • Chewy texture and rich flavor • Ideal for barbecues or as bar snacks	Korea	Foodjang, 2017
		Chicken hearts	• Whole, unseasoned chicken hearts • Uniform size for easy skewering and grilling	US	TC.FARM, 2023
	Liver	Chicken liver *yakitori*	• Popular Japanese item • Chicken liver grilled with sweet soy sauce • Served as a *yakitori* skewer	Japan	Ono & Salat, 2011
		Citarella chopped chicken liver spread	• Chicken liver-based spread • Soft texture and rich flavor	US	Citarella, 2015
		*Foie de volaille confits*	• French-style chicken liver confit • Slow-cooked in duck fat with herbs and spices • Enhanced flavor and tender texture	France	Carrefour, 2022
		Chicken liver pâté	• American-style reinterpretation of European *pâté* • Served on crackers or baguettes with wine or as a brunch snack • Noted for its rich, deep flavor	US	Schallerweber, 2025
		Organic chicken liver sausage	• Organic chicken liver sausage with chicken meat • Seasoned with herbs and Celtic sea salt • Mild, clean flavor without artificial additives	Netherland	Dewoeste grond, 2025
	Skin	Chicken skin snack	• Crispy fried chicken skin snack • High-protein, low-carbohydrate product • Premium beer snack	EU	Chickencrackling, 2022
		Fried chicken skin	• Ready-to-eat after reheating in an air fryer or frying pan • Convenient instant food option	Korea	Jinji, 2025
Others	Sheep heart	Lamb heart	• Raw sheep heart • Rich in vitamin B12 and omega-3 fatty acids	US	Togetherfarms, 2024
	Sheep liver	Lamb liver	• Refrigerated distribution for home cooking • Rich in vitamin A	EU	Sainsbury, 2018
		Skilpadjies	• South African dish with minced lamb liver • Seasoned and wrapped in caul fat • Grilled for a rich, crispy flavor	South Africa	Unclebeef, 2021
	Sheep stomach	Tripous	• Sheep’s stomach stuffed with pork intestines, ham, and parsley • Slow-cooked with white wine and spices for enhanced flavor	France	Lanaucelloise, 2015
	Sheep tongue	Lamb’s tongue tacos	• Easy-to-cook lamb tongue with tender, mild flavor • Ideal for shredding and seasoning • Pairs well with salsa and sour cream in tacos	EU	Finefoodspecialist, 2019
	Duck feet	Duck feet	• Frozen and versatile • Suitable for spicy preparations and various dishes.	Asia	Kimmart, 2025
	Duck gizzard	Duck confit gizzards	• Low-temperature dried American duck stomach jerky sticks • Convenient, ready-to-eat snack form	US	Bella Bella Gourmet, 2025
	Duck liver	Duck liver	• Ready for use in a variety of dishes	EU	Reatonfood, 2025
				Singapore	Kendo, 2021
	Duck tongue	Sweet spicy duck tongue	• Frozen duck tongue seasoned with assorted sauces and spices • Easy to cook and adaptable to many dishes	Asia	XIAO HU DUCK, 2023

Data adapted from multiple sources and refer to supplementary information

The kidneys and liver are particularly rich in trace elements and minerals, with the liver also serving as a significant source of vitamins. Animal fats, such as lard and tallow, are high-energy sources used in both food products and animal feed (Jayathilakan et al., 2012; Toldrá et al., 2012). Gelatin extracted from animal bones and skin is widely used as a food ingredient, while endocrine glands—including the pituitary, thyroid, thymus, adrenal glands, ovaries, testes, and pancreas—are valuable for producing medical enzymes and hormones (Awan et al., 2015; Salem et al., 2023). Due to their excellent nutritional content, many visceral by-products are suitable for use as additives or reprocessed materials in meat products. For example, bovine bone extracts provide protein, vitamins, minerals, and collagen, and are used as food additives. The head is processed into head cheese, the liver is used in products like chicken nuggets, and the stomach features in traditional Mexican dishes such as *menudo* (Table 1). Cooked beef tongue is commonly sliced and consumed directly or used as a topping for sandwiches or salads, or as an ingredient in broths (Table 1). Pork by-products are commonly found in traditional foods such as blood sausage and black pudding, and are also used in broths, hamburger patties, and Spanish dishes like *chicharrón* (Table 1). In poultry, the skin and heart are often grilled or served as snacks, while the liver is incorporated into sausages or spreads, particularly in European markets (Table 1).

Beyond their culinary uses, slaughter by-products are valuable sources of bioactive compounds. For example, bones from cattle and pigs serve as sources of protein, gelatin, and hydroxyapatite; the liver provides ferritin; the lungs yield protein concentrates; and the hide is a key source of collagen for food and pharmaceutical applications (Table 2). In chickens, blood yields blood corpuscle hydrolysate, the comb provides hyaluronic acid, feathers supply hydrolyzed feather protein or keratin, and skin and cartilage yield collagen or gelatin. The viscera also provide extractable protein materials (Table 2). In goats, the femur serves as a source of mesenchymal stem cells and hydroxyapatite; hooves provide keratin; and tendons yield collagen. In ducks, chenodeoxycholic acid (CDCA) and red blood cell hydrolysate can be extracted from the gallbladder and blood, respectively (Table 2). Collectively, these by-products represent important raw materials for the development of high-value bioactive substances.

**Table 2:** Biological materials derived from animal slaughter by-products and their applications

Animals	By-products	Products	Purpose of product	Characteristics	Reference
Cattle	Bone	Protein	Meat additives	• Bovine bone-extracted protein for finely ground sausages • Texture comparable to commercial protein-based products • Stress similarity enabling substitution of non-meat proteins	Boles et al., 2000
		Gelatin	Constituent in food, medicine, pharmacy, photography, and cosmetic	• Low molecular weight peptides (16–150 kDa) in gelatin with essential amino acids • Actinidin enzyme processing enhancing yield, gelatin strength, and viscosity • α-chain and peptide presence improving bovine skin gelatin properties	Ahmad et al., 2019
		Hydroxyapatite	Biomaterials development	• Exhibits biocompatibility for medical applications • Suitable as coating material for bone implants • Applicable in tissue engineering	Said et al., 2024
	Liver	Ferritin	Functional properties (e.g., iron supplement)	• Hydrophilic and thermostable protein regulating iron metabolism • Mediates redox reduction of iron	Song et al., 2024
	Lung	Protein concentrates	Emulsifiers	• High emulsifying activity with superior surface hydrophobicity • Lower surface and interfacial tension compared to most commercial protein ingredients	Darine et al., 2010
	Skin	Collagen	Collagen-derived applications	• Increased collagen content using modified acid-enzyme solubilization • Clean, white, agglomerated powder resembling commercial standard collagen	Noorzai et al., 2020
Pig	Blood	Haemoglobin hydrolysates	Natural antioxidants	• Protein-rich porcine blood hydrolysate with natural antioxidant properties • Higher DPPH radical scavenging activity than native hemoglobin • Amino acid type/sequence effects on antioxidant activity requiring further study	Chang et al., 2007
	Bone	Protein hydrolysate	Nutraceuticals, animal feed, and functional food	• Protein hydrolysates produced via Alcalase enzymatic hydrolysis • High hydrolysis degree of 53.3 ± 5.1% • Abundance of low molecular weight peptides (< 1.2 kDa) • Demonstrated strong antioxidant activity in ABTS and ORAC assays	Borges et al., 2022
		Hydroxyapatite	Biomedical applications such as bone grafts or tissue engineering	• Hydroxyapatite production via 700 °C calcination of solid residues • Nano-sized crystals with Ca/P ratio of 1.65 (near theoretical 1.67) • High-purity material for biomedical applications like bone grafts and tissue engineering	
	Liver	Zinc-protoporphyrin IX (ZnPP)	Nitrite substitute	• Porcine liver’s optimal pH for ZnPP formation • Enhanced meat product color via ZnPP addition • Application in meat products requiring further research.	Wakamatsu et al., 2015
		ZnPP	Meat protein sources and bioactive peptide generating	• ZnPP-enhanced meat color as nitrite substitute • Homeostatic liver hydrolysate from ZnPP extraction • Antioxidant and antihypertensive properties in hydrolysate	López-Pedrouso et al., 2023
	Skin	Collagen	Polyfunctional food-grade biopolymer	• Gelatin from leather enhancing gelation and dietary fiber content • Increased hydrophilicity aiding food matrix formation and water binding • Denser, more uniform meat jelly product	Gorlov et al., 2018
	Spleen	Functional protein	Emulsifiers, texturizing agents and protein replacers	• Spleen-derived proteins with high heme iron content and bioavailability • Easy absorption and potential as emulsifier, texturizer, or protein substitute	Toldrà et al., 2019
	Organs (liver, lung, heart, stomach, small intestine and large intestine)	Heparin	Anticoagulant and potential drug applications	• Primary heparin extraction from liver • Anticoagulant, antihypertensive, and antibacterial activity against Staphylococcus aureus • Disease prevention and inhibition applications	Lee et al., 2020
	Organs (colon, appendix, rectum, pancreas, heart, liver, and lung)	Organ hydrolysates	Antioxidants	• Antioxidant capacity in hydrolyzed organ extracts (colon, liver, appendix) • Lipid oxidation inhibition through organ hydrolysates	Damgaard et al., 2014
Chicken	Blood	Blood corpuscle hydrolysate	Functional food with antihypertensive effect	• Blood hydrolysate produced via Alcalase enzymatic hydrolysis • Demonstrated antihypertensive properties • Rich source of amino acids such as phenylalanine (F), glycine (G), alanine (A), valine (V), isoleucine (I), and leucine (L)	Wongngam et al., 2020
	Crest	Hyaluronic acid (HA)	For use in cosmetics, pharmaceuticals, and functional foods	• HA extraction from chicken crests via enzymatic hydrolysis/precipitation • 14.9 µg hexuronic acid per mg dried tissue with strong DPPH radical scavenging activity • Validation of poultry by-products as functional biomaterials	Rosa et al., 2008
	Feathers	Hydrolyzed feather protein	Feed protein source	• Enhanced digestibility and minimized amino acid loss via NaOH/Na₂SO₃ hydrolysis with autoclaving • Residual fractions enriched in cystine and lysine for high-quality feed applications • Soluble fractions containing histidine and methionine suitable for cat food formulations	Adler et al., 2018
	Feathers	Keratin	Creating sustainable packaging by recycling chicken feather waste.	• Enhanced tensile strength and surface smoothness with increasing chicken feather keratin content • Reduced transparency and solubility in developed biocomposite films • Over 50% soil degradation observed within 12 days • Sustainable packaging potential utilizing poultry by-products	Oluba et al., 2021
	Skin	Collagen	Various industries such as cosmetics, pharmaceuticals, and food additives	• Collagen from chicken skin retains triple-helical structure for biocompatibility and functional versatility • Rich in collagen-specific amino acids (glycine, proline, hydroxyproline) • Potential applications in biomedical and food industries utilizing poultry by-products	Matinong et al., 2022
	Sternum cartilage	Collagen	Cartilage formation and regeneration	• Adipose-derived stem cells (ASCs) combined with chicken cartilage-derived type II collagen hydrogel and fibrin sealant • Effective promotion of cartilage repair through synergistic biomaterial interaction • Utilization of poultry-derived collagen for biocompatible tissue engineering applications	Lazarini et al., 2017
	The mechanical separation of chicken meat (W-MSM)	Bioactive compounds	A source of high-value dietary supplements	• Papain-hydrolyzed W-MSM yielding HA, chondroitin sulfate (CS), peptides, and amino acids • CS and HA as key bioactive components for cartilage regeneration mechanisms • Versatile applications in animal supplements, functional foods, and pharmaceutical formulations	Stiborova et al., 2020
	Viscera	Enzymatic hydrolysis of chicken viscera protein concentrate (CVPC)	Developing value-added materials from bioactive peptides.	• Highest bioactivity achieved with 1:1:1 Alcalase^®^ 2.4 L/Flavourzyme^®^ 500 L/Neutrase^®^ 0.8 L hydrolysis • Non-fractionated hydrolysate outperforming ultrafiltered fractions in bioactive properties • Synergistic enzyme action enhancing protein functionality for antioxidant or bioactive applications	dos Santos Aguilar et al., 2020
Black bone chicken	Feet and skin	Gelatins from black-bone chicken feet and skin (BCFG and BCSG)	Alternative source of production of gelatin with good physicochemical properties	• Enhanced gel strength in BCFG and BCSG compared to commercial bovine gelatin (BG) • Increased NaOH concentration reduces thermal stability and gel strength in BCSG • Optimal alkaline treatment conditions required for maintaining protein functionality	Saenmuang et al., 2020
Others	Femurs of sheep and goat	Mesenchymal stem cells (MSCs)	Isolation of multipotent cells for tissue engineering and regeneration.	• Isolation of MSCs from sheep and goat long bone marrow via adherent cell culture • Selective expansion of adherent cells yielding high MSC quantities with standard surface marker expression • Demonstrated multi-lineage differentiation capacity in isolated mesenchymal stem cells	Santra et al., 2017
	Femur of sheep	Hydroxyapatite	Biomaterial for application in bone regeneration	• Enhanced hydroxyapatite structural stability and electrical charge storage capacity through MgO addition at varying concentrations • Sheep femur-derived bone graft material effectiveness improved via magnesium oxide integration • Promoted cell growth and bone regeneration through optimized charge properties	Gavinho et al., 2023
	Hoof of goat	Keratin	Biomaterials for tissue regeneration	• Modified Shindai solution enabling high-molecular-weight keratin extraction from goat hooves • Retention of structural integrity and promotion of cell adhesion/proliferation in extracted keratin • Potential biomedical applications leveraging keratin’s bioactive properties	Kumar et al., 2017
	Small intestine submucosa (G-SIS) of goat	Decellularized goat small intestine submucosa (DG-SIS)	Biomaterial development for tissue engineering and regeneration.	• DP4-optimized biomaterial achieves effective cellular component removal while preserving small intestine submucosa ECM • Residual DNA reduced to 50 ng/mg for minimized immune response risk • High GAG/proteoglycan retention supports cell interaction, 3D tissue structure maintenance, and hydration	Singh et al., 2022
	Tannery of sheep and goat	Hydrolyzed protein of delimed tannery fleshings (TF)	Animal feed supplements and antioxidants	• Organic acid hydrolysis and Enterococcus faecium fermentation enhance proline, tyrosine, valine, and leucine content in TF hydrolysate, correlating with antioxidant efficacy. • Antimicrobial and antioxidant potential positions this hydrolysate as a sustainable animal feed protein source for reducing oxidative stress in livestock	Balakrishnan et al., 2011
	Tendon of goat	Collagen	Biomaterials for skin tissue engineering	• Goat tendon collagen extracted via 1% acetic acid treatment, composed of type I collagen structure • Promotes cell adhesion and growth with low immune response in murine models • Demonstrates wound healing efficacy through enhanced tissue regeneration mechanisms	Banerjee et al., 2012
	Viscera (liver, lungs, heart) of goat	Protein hydrolysates	Protein-rich materials for health-focused applications.	• Protein hydrolysates from Alcalase^®^ and Brauzyn^®^ show excellent solubility, emulsifying ability, and antioxidant activity • Rich in essential amino acids, including lysine, phenylalanine + tyrosine, leucine, and methionine • Suitable for food applications requiring functional and bioactive protein ingredients	de Queiroz et al., 2017
	Bile of duck	Chenodeoxycholic acid (CDCA)	Medical treatment of metabolic diseases	• CDCA utilized for anti-inflammatory effects and cholesterol gallstone dissolution in medical treatments • Calcium salt-based extraction optimized with hydrogen peroxide, methyl alcohol, glacial acetic acid, and calcium chloride • 30% yield achieved through refined extraction protocol	Hu et al., 2018
	Blood of duck	Red blood cell hydrolysate (RBCH)	New functional antioxidant food additive.	• RBCH contains > 50% essential amino acids, meeting adult daily requirements except isoleucine • Demonstrates superior solubility, emulsification, foaming properties, and antioxidant activity • Versatile applications as high-value food material, functional additive, and bioactive biomaterial	Zheng et al., 2018

Data adapted from multiple sources and refer to supplementary information

### Slaughter blood

Slaughter blood is a significant by-product of the meat industry, accounting for approximately 4–5% of live body weight in cattle and 3–5% in pigs (Chiroque et al., 2023). It is rich in high-quality protein and contains essential nutrients, including all major amino acids and highly bioavailable heme iron (Sorapukdee & Narunatsopanon, 2017).

Blood consists of two main components: blood cells (20–40%) and plasma (60–80%). Blood cells include red and white blood cells, as well as platelets, while plasma is composed of functional proteins—albumin (50–60%), globulin (40%), and fibrinogen (0.6%) (Tarté, 2011; Nazifa et el., 2021). On average, whole blood comprises 80–82% water, 18–20% total solids, 13–15% protein, and less than 1% fat and carbohydrates. These components are used in various industrial applications as stabilizers, clarifying agents, and emulsifiers (Toldrà et al., 2019; Chiroque et al., 2023). Furthermore, freeze-dried blood contains trace minerals such as iron, magnesium, zinc, copper, manganese, and chromium (Sorapukdee & Narunatsopanon, 2017), further highlighting its nutritional and functional value.

Due to its rich nutritional and bioactive properties, animal blood has attracted considerable interest for industrial applications. The main nutrient compositions of blood from major livestock species are summarized in Table 3. While some variation exists among cattle, pigs, and chickens, their overall blood profiles remain largely comparable (Table 3), reflecting the evolutionary conservation of key components essential for vertebrate survival. However, the concentrations of specific blood constituents may vary depending on factors such as species, breed, sex, age, and diet (Xuan et el., 2018; Lee et el., 2024).

**Table 3:** Major blood components in domestic animals (cattle, pigs, and chickens)

Component		Cattle	Pigs	Chickens
Cellular	Platelets (10^9^/L)	254.0–441.9	190–400	-
	Thrombocytes (10^9^/L)	-	-	7.1–88.3
	Red blood cells (10¹²/L)	7.1–7.8	5.5–9.2	1.8–4.4
	White blood cell (10^9^/L) (B lymphocytes, Basophils, Eosinophils, Monocytes, Neutrophils, T lymphocytes)	7.2–9.5	9.4–30	1.0–10.2
Carbohydrates	Glucose (mmol/L)	1.7–3.9	3.8–7.2	6.2–27.5
Proteins	Albumin (g/L)	28.1–41.1	33.7–41.9	13.2–24.0
	ALP (g/L)	119.8–121.2	46.3–76.7	9.8–11.1
	ALT (U/L)	13.6–24.9	25.0–30.3	28.7–53.7
	AST (U/L)	36.2–37.7	24.0–29.7	176.6–219.0
	Globulin (g/L) (Alpha-globulin, Beta-globulin, Gamma-globulin)	32.0–47.6	12.7–34.8	9.3–29.3
	Hemoglobin (g/L)	114.7–124.0	104.5–111.5	71.0–181.8
	Total protein (g/L)	63.0–81.0	46.3–76.7	22.5–65.0
	Other proteins (Fibrinogen, LDH, Thrombin)	-	-	-
Lipids	Cholesterol (mmol/L)	2.9–3.4	2.1–2.5	2.7–3.9
	Lipoprotein (HLDL, LDL, VLDL)	-	-	-
	Triglycerides	-	-	-
Minerals	Calcium (mmol/L)	2.5–3.5	0.9–2.4	2.3–6.8
	Phosphorus (mmol/L)	1.7–2.5	2.4–3.4	1.3–2.2
	Other minerals (Chlorine, Copper, Iron, Magnesium, Manganese, Potassium, Sodium, Zinc)	-	-	-
Metabolites	Bilirubin (µmol/L)	6.6–8.3	0.8–1.0	1.2–1.7
	Urea (mmol/L)	4.7–8.6	1.7–7.2	1.0–1.2
	Other metabolites (Creatinine, Lactate, Uric acid)	-	-	-

Data adapted from multiple sources and referred to supplementary information. ALP, alkaline phosphatase; ALT, alanine aminotransferase; AST, aspartate aminotransferase; LDH, lactate dehydrogenase; HLDL, High-density lipoprotein; LDL, Low-density lipoprotein; VLDL, Very low-density lipoprotein

Studies have shown that cattle exhibit the highest levels of total protein, alkaline phosphatase, globulin, hemoglobin, and urea, followed by pigs and chickens (Table 3). In contrast, levels of alanine aminotransferase (ALT), aspartate aminotransferase (AST), and calcium are highest in chickens—likely reflecting their calcium-enriched diets for egg production. Moreover, the blood of cattle and pigs has been reported to exhibit functional properties, including angiotensin I-converting enzyme (ACE) inhibitory activity, antioxidant effects, antimicrobial properties, and opioid-like activity (Bah et al., 2013). Notably, hemoglobin has been shown to serve as a precursor for high-value bioactive compounds such as antioxidant peptides, antimicrobial agents, ACE inhibitors, and heme iron–polypeptide complexes (Cheng et al., 2024).

As discussed earlier, interspecies differences in physiology lead to inherent variability in blood composition and biochemical profiles, complicating direct comparisons. Nevertheless, research on the systematic characterization and targeted valorization of blood components remains limited—particularly concerning the functional properties and industrial applicability of individual components. This is because blood composition can vary substantially depending on species, region, and feeding conditions, and such variability constitutes a critical challenge in ensuring compositional consistency and reproducibility for industrial applications. Moreover, the difficulties associated with obtaining large-scale samples and conducting pooled analyses further constrain research aimed at practical industrial implementation. In addition, given that animal blood may contain numerous unidentified bioactive compounds, comprehensive analytical profiling and in-depth studies are essential to fully harness its potential for future applications.

## Research on cell culture

The first successful animal cell culture was performed in 1907 by Ross Granville Harrison, who cultured nerve fibers from frogs on a coverslip. In 1961, Mauro identified and cultured satellite cells for the first time using electron microscopy while examining the femoral muscle of adult frogs. The subsequent development of genetically defined mouse strains further advanced mammalian cell culture and facilitated the establishment of various cell lines, significantly contributing to biomedical and cancer research (Arango et al., 2013).

Cultured meat products will inevitably be composed of multiple cell types. This will likely require muscle satellite cells, myoblasts, myocytes, adipose-derived stem cells, adipocytes, and fibroblasts (O’Neill et al., 2021). Among these, muscle satellite cell culture, considered the most important for cultured meat production, made a significant advance in the study of muscle development in 1974 when Bischaff formally isolated muscle satellite cells from adult rat skeletal muscle via enzymatic digestion (Doumit & Merkel, 1992; Anderson & Pilipowicz, 2002). Currently, satellite cells are typically harvested via muscle biopsy, followed by a series of processes including disinfection, mechanical and enzymatic digestion, filtration, and centrifugation to yield a viable cell population for culture (Lee et al., 2021). Given the limited number of cells obtained from initial isolation, subculturing is necessary to expand the cell population for downstream applications. During subculture, tailored media formulations and additives are used to promote either cell proliferation or differentiation, depending on experimental objectives (Freshney, 2006).

However, primary non-immortalized cells experience progressive declines in proliferative capacity and cellular function with increasing passage number, primarily due to telomere attrition. Telomeres, which are repetitive nucleotide sequences at chromosome ends, preserve genomic stability. Their shortening leads to cellular senescence, loss of DNA repair capacity, and cessation of proliferation (Mastromonaco et al., 2006). Therefore, the application of muscle satellite cells in cultured meat production necessitates either repeated tissue collection or the development of immortalized cell systems. Recent studies have developed induced pluripotent porcine stem cells capable of over 220 doublings and immortalized bovine satellite cells capable of over 120 doublings (Ezashi et al., 2009; Stout et al., 2023). The U.S. Food and Drug Administration (FDA) has approved the production of cultured chicken using telomerase reverse transcriptase (TERT)-immortalized fibroblasts (FDA Center for Food Safety and Applied Nutrition, 2022). However, the use of genetically modified or transformed cell lines in food products warrants rigorous safety assessments and ethical considerations.

Cell cultures are generally categorized into three main types: primary cells, self-renewing (stem) cells, and transformed (immortalized) cells (Segeritz & Vallier, 2017). Primary cells are directly isolated from human or animal tissues and are relatively easy to obtain. However, their proliferation potential is limited, often requiring continuous sourcing for sustained experiments. Handling of primary cells must adhere to strict biosafety and ethical standards due to concerns regarding tissue origin and use. Myoblasts and satellite cells, first identified by Mauro (1961), are examples of primary cells capable of differentiating into myofibers and are widely employed in skeletal muscle tissue engineering (Post, 2012). To ensure high cell purity, techniques such as magnetic-activated cell sorting (MACS) and fluorescence-activated cell sorting (FACS) are used. MACS involves labeling target cells with antibodies bound to magnetic beads for separation (Pan & Wan, 2020), while FACS utilizes flow cytometry to sort fluorescently labeled cells (Liao et al., 2016). However, these methods may reduce cell viability and introduce risks of genetic alteration, necessitating cautious application (Basu et al., 2010).

Self-renewing cells, commonly referred to as stem cells, are characterized by their capacity for both self-renewal and differentiation (McKee & Chaudhry, 2017). Stem cells can give rise to over 200 different cell types and are broadly categorized into embryonic stem cells (ESCs), induced pluripotent stem cells (iPSCs), and mesenchymal stem cells (MSCs). MSCs, typically isolated from bone marrow or adipose tissue, can differentiate into a limited range of lineages, including osteocytes, chondrocytes, and adipocytes (Pittenger et al., 1999; McKee & Chaudhry, 2017). These cells are generally cultured using adherent methods, and after subculturing, can be directed toward specific lineages by stimulating relevant target genes (NIH, 2016). Beyond research applications, adult stem cells have also demonstrated clinical utility in the treatment of leukemia, burns, and corneal damage (Kalra & Tomar, 2014).

Transformed cells are genetically modified or naturally derived immortalized cells that exhibit indefinite proliferation and support long-term culture. These cells originate from tumor tissues or are artificially modified using viral vectors, chemical treatment, or electroporation to bypass senescence (Soice & Johnston, 2021). Due to their robust growth characteristics, transformed cells are widely used in research involving cell and tissue engineering, drug screening, and cellular differentiation (Maqsood et al., 2013; Voloshin et al., 2023). However, extended culture can lead to genomic and phenotypic instability, along with potential loss of cellular function (Kaur & Dufour, 2012). Additionally, concerns over contamination and the use of human-derived cell lines continue to raise ethical and safety issues (Meslin & Quaid, 2004).

Most cell cultures rely on basal media supplemented with FBS, antibiotics (e.g., penicillin–streptomycin, Gentamicin), and growth factors such as basic fibroblast growth factor (bFGF), granulocyte/macrophage colony-stimulating factor (GM-CSF), transforming growth factor beta (TGF-ß), interleukin-1 (IL-1), insulin-like growth factor (IGF-1), insulin, and hydrocortisone to support cellular growth and function (Whitford, 2005; Hassan & Ahmad, 2020). The choice of medium depends on the specific requirements of the cell type and research application. Commonly used media include Dulbecco’s Modified Eagle’s Medium (DMEM), Ham’s F-12, and RPMI 1640. Table 4 presents a summary of the nutritional composition of the key components used in cell culture, including basal media, FBS, antibiotics, and additives. Recently, increasing attention has been given to the development of nutrient-based additives and the optimization of culture conditions to enhance cell viability, proliferation, and differentiation. These efforts are crucial for improving the scalability and efficiency of cultured cell systems, particularly in emerging applications such as tissue engineering and cultured meat production.

**Table 4:** Common reagents used in cell culture

Reagent type	Components
Basal media (e.g., Minimum Essential Medium (MEM), Dulbecco’s Modified Eagle Medium (DMEM), Iscove’s Modified DMEM, Ham’s F12, Medium 199, RPMI 1640, Leibovitz’s L-15, etc.)	**Sugar**: Glucose **Salts**: Calcium chloride, Potassium chloride, Magnesium sulfate, Sodium chloride, Sodium phosphate, Sodium bicarbonate **Amino acids**: L-Arginine Hydrochloride, L-Cystine 2HCL, L-Glutamine, L-Histidine hydrochloride-H2O, L-Isoleucine, L-Leucine, L-Lysine Hydrochloride, L-Methionine, L-Phenylalanine, L-Threonine, L-Tryptophan, L-Tyrosine Disodium salt dihydrate, L-Valine **Vitamins**: Thiamine (Vitamin B1), Riboflavin (Vitamin B2), Nicotinamide (Vitamin B3), Pantothenic Acid (Vitamin B5), Pyridoxine (Vitamin B6), Folic Acid (Vitamin B9), Choline, Myo-Inositol (Vitamin B8)
Fetal Bovine Serum (FBS)	**Biochemicals**: Alkaline phosphatase, ALT, AST, Bicarbonate, Bilirubin, BUN, BUN/Creatinine ratio, Calcium, Chloride, Cholesterol, Creatinine, GGPT, Glucose, HDL, Iron, Iron saturation, LDH, LDL, Phosphorus, Potassium, Sodium, TIBC, Triglycerides (TG), Uric Acid **Hormone**: Estradiol, Insulin, Progesterone, Testosterone, Thyroxine (T4)
Antibiotics	Penicillin, Streptomycin, Gentamicin, Amphotericin, etc.
Supplements	**Growth factor**: IGFs, insulin, TGF-β, FGF, PDGF, HGF **Hormone**: Thyroid hormones, Testosterone, Glucocorticoids **Protein**: Creatine, Heme Proteins, Transferrin, BMP, mTOR

ALT: alanine aminotransferase, AST: aspartate aminotransferase, BUN: blood urea nitrogen, GGPT: gamma-glutamyl transpeptidase (or GGT, gamma-glutamyl transferase), HDL: high-density lipoprotein, LDH: lactate dehydrogenase, LDL: low-density lipoprotein, TIBC: total iron binding capacity, IGFs: insulin-like growth factors, TGF-β: transforming growth factor-beta, FGF: fibroblast growth factor, PDGF: platelet-derived growth factor, HGF: hepatocyte growth factor, BMP: bone morphogenetic protein, mTOR: mammalian target of rapamycin

### Fetal bovine serum (FBS) in cell culture

FBS is the most widely used supplement in cell culture, valued for its comprehensive mix of hormones, growth factors, amino acids, proteins, vitamins, minerals, trace elements, carbohydrates, and lipids (Van der Valk et al., 2018; Lee et al., 2022, 2023). Derived from the fetuses of pregnant cows, FBS contains low levels of immunoglobulins, which reduces the likelihood of immune rejection compared to sera obtained from adult animals (Lee et al., 2022).

FBS provides essential growth factors absent in standard sera, such as epidermal growth factor (EGF), fibroblast growth factor (FGF), nerve growth factor (NGF), IGF, platelet-derived growth factor (PDGF), and TGF, all of which are critical for promoting cell proliferation (Lee et al., 2022). Additionally, cytokines present in FBS support cell growth and function; media containing FBS have been shown to induce the secretion of cytokines such as IL-6 and IL-8 (Lee et al., 2022; Santos et al., 2023). FBS also contains various hormones, including insulin, progesterone, testosterone, and thyroxine—among which insulin and growth hormone are particularly effective at enhancing cell proliferation (Yao & Asayama, 2017). The trace levels of minerals and vitamins in FBS further contribute to cell proliferation and differentiation (Lee et al., 2022). Owing to this diverse and bioactive composition, FBS remains a critical component in most current cell culture systems, supporting proliferation, differentiation, and functional maintenance of a wide variety of cell types.

FBS is typically collected from bovine fetuses over three months of gestational age, as younger fetuses possess underdeveloped hearts that complicate blood collection. After the dam is slaughtered, the fetus is aseptically removed, sterilized, and subjected to cardiac puncture, where a needle is inserted between the ribs to extract blood directly from the heart. The collected blood is transferred into sterile vacuum tubes, coagulated at low temperatures, and centrifuged under refrigerated conditions to obtain high-purity serum. This process is usually completed within 30 min and yields approximately 150 mL of raw FBS. Final processing includes 0.1 μm membrane filtration and aseptic filling to ensure product sterility and consistency (Jochems et al., 2002; Lee et al., 2022; Sartorius, 2024).

However, the production of FBS raises significant ethical and practical concerns. An estimated one million fetal calves are required annually for global FBS production (Van der Valk et al., 2004). Following maternal death, fetuses experience acute oxygen deprivation, and there is a possibility that they may retain some level of consciousness during blood collection (Jochems et al., 2002). This fundamentally infringes upon the life potential of bovine fetuses that could otherwise have grown and developed, and as the demand for FBS increases, the number of sacrificed fetuses rises exponentially. In this process, both the fetus and the dam are lost, further compounding the opportunity costs. Such realities not only account for the high price of FBS but also provoke profound ethical controversy, as they involve sacrificing calves before birth, ultimately reinforcing negative perceptions among consumers. Additionally, variability in serum composition due to seasonal and geographical factors can affect experimental reproducibility, and the use of animal-derived products introduces risks of microbial or viral contamination (Van der Valk et al., 2010). As of 2025, the commercial cost of FBS is approximately 700,000 KRW per 500 mL, prompting growing demand for serum-free and chemically defined media. Consequently, research into ethical and cost-effective FBS alternatives is intensifying worldwide.

### FBS substitutes and utilization of slaughter by-products

Research into alternatives to FBS has been ongoing since the 1950 s, with initial efforts focusing on replacing individual serum components. Current strategies for reducing FBS dependency include optimizing existing media compositions, lowering serum concentrations, and developing fully serum-free formulations (Brunner et al., 2010). Concerns about FBS center on supply instability, high cost, and the risk of contamination with pathogens such as viruses, driving intensified efforts to establish serum-free alternatives (Chelladurai et al., 2021). Despite several decades of research and development, a definitive substitute capable of completely replacing FBS has yet to be established (Van der Valk et al., 2010; Chelladurai et al., 2021). A principal reason for this limitation is that the precise and complete composition of FBS remains incompletely defined (Chelladurai et al., 2021). Furthermore, even in approaches that avoid the direct use of FBS, virtually all serum alternatives still necessitate supplementation with animal-derived constituents, including growth factors, albumin, and other bioactive molecules.

The earliest serum-free medium was developed to replace cerebrospinal fluid for neuronal cell culture and has since been adapted for cardiomyocytes, motor neurons, myocytes, and various other cell types (Van der Valk et al., 2018). FBM™, TesR™, and Essential 8™ (E8) are serum-free media for human PSC and fibroblast cultures, utilizing recombinantly expressed growth factors and other essential proteins (Kolkmann et al., 2020; Skrivergaard et al., 2023). Furthermore, E8 media was further optimized for a weekend-free induced pluripotent stem cell (iPSC) culture (renamed B8), containing a simple mixture of glucose, amino acids, vitamins, salts and fatty acids, minerals, and proteins, leading to further research as a serum-free medium for cell-cultured meat. The addition of recombinant rice-derived albumin to B8 media (renamed Beefy-9) enabled sustained proliferation of bovine muscle satellite cells over seven passages, although this did not reach the levels observed in conventional FBS-supplemented growth media (Kuo et al., 2020; Stout et al., 2022).

In addition to serum-free media, various animal- and plant-derived substances have been studied as FBS replacements over the past several decades. For instance, soybean meal fermented with *Aspergillus oryzae*, *Bacillus subtilis*, or *Bacillus licheniformis* has been used in porcine muscle stem cell culture (Kim et al., 2023). Alkaline-treated hydrolysates from brown mealworms replaced up to 50% of FBS at 0.1% concentration. Similarly, enzyme extracts from pea, algae, and mushrooms at 1–2.5% concentrations maintained skeletal density in zebrafish embryonic stem cells at levels comparable to 10% FBS (Amirvaresi & Ovissipour, 2024). Protein extracts from *Auxenochlorella pyrenoidosa* promoted goldfish muscle cell proliferation, while chlorella protein extracts enhanced *Myf5* gene expression, facilitating myoblast fusion and differentiation (Punch et al., 2009; Dong et al., 2024).

Plant-derived commercial supplements such as Prolifix have also shown promise in epithelial cell proliferation. However, although 10% Prolifix supplementation supported cell growth, it resulted in slower proliferation and diminished cryopreservation capacity compared to FBS, highlighting the need for further optimization (Van der Valk et al., 2004).

Several animal-derived proteins have also been explored. Human serum albumin (HSA), an ingredient approved by the U.S. Food and Drug Administration (FDA), promoted C2C12 and baby hamster kidney (BHK) cells at 1% supplementation (De Castro et al., 2006). Sericin, a protein derived from silkworm cocoons, promoted cell proliferation at 30 µg/mL, with effects comparable to or exceeding those of FBS (Liu et al., 2016). Similarly, media supplemented with 20% buffalo ocular fluid—rich in growth factors, hypoxanthine, and fibronectin—supported cell cryopreservation and recovery, making it suitable for serum blends (Varma et al., 2015).

Earthworm coelomic fluid, containing 70–80% protein and amino acids, supported effective cell growth at 10% supplementation (Vasantha et al., 2019). Glucose and amino acids extracted from microalgae (5–20%) significantly increased C2C12 cell viability (Okamoto et al., 2020), while ethanol extracts of egg white (10%) effectively promoted the proliferation of chick satellite cells (Lee et al., 2024a). Autologous serum has been successfully used to culture menstrual blood-derived mesenchymal stem cells, maintaining CD73 and CD105 expression and supporting FBS-free culture (Rezaei Kiasari et al., 2024).

To address the biological variability of FBS, human umbilical cord serum has been used to culture human amnion-derived stem cells, maintaining the expression of pluripotency and proliferation genes OCT-4 and Rex-1, with proliferation efficacy comparable to that of FBS (Kim et al., 2007). Additionally, recombinant mixtures of growth factors and cytokines (IGF-I, IGF-II, bFGF, LIF, TGF-β1, and GM-CSF), combined with recombinant albumin and hyaluronic acid, have been shown to effectively support cell proliferation and embryo development (Moreno et al., 2015).

Human platelet lysates and platelet-derived factors are also gaining traction in FBS replacement research. Platelet α-granules release several growth factors, including PDGF, EGF, VEGF, bFGF, HGF, and TGF-β1, which contribute to cell attachment, proliferation, and wound healing (Rauch et al., 2011; Van der Valk et al., 2018; Duarte Rojas et al., 2024).

Bioactive substances—non-essential components derived from plant, animal, and marine sources—can modulate human biochemical and physiological functions (Bah et al., 2013). Bioactive peptides, consisting of 2–50 amino acids, are particularly attractive due to their high purity, ease of synthesis, scalability, and stability (Hernández-Ledesma et al., 2011; Dayem et al., 2023). These peptides enhance cell adhesion, scaffold binding, proliferation, and differentiation. For example, peptides obtained from trypsin/chymotrypsin-based hydrolysis of whey protein effectively promoted immune cell proliferation (Mercier et al., 2004), and bovine β-lactoglobulin hydrolysates enhanced mouse splenocyte proliferation (Mahmud et al., 2004). Similarly, bovine lactoferrin hydrolysates induced mouse spleen lymphocyte proliferation (Pi BingBing et al., 2018). Bioactive peptides also support osteogenic differentiation through SMAD phosphorylation and Wnt signaling pathways (Ko et al., 2013; Lukasova et al., 2017)d cadherin mimetic peptides encapsulated in hydrogels have been shown to enhance chondrogenic differentiation of human mesenchymal stem cells (Mohammed et al., 2021).

Although research into FBS alternatives derived from slaughterhouse blood and by-products is still in its early stages and is mostly limited to adult serum, some promising results have been reported. Bovine serum has demonstrated the potential to replace FBS in various cell lines, including primary mouse astrocytes, primary human fibroblasts, L6 cells, and umbilical cord mesenchymal stem cells (Yu et al., 2013; Shehzadi et al., 2024). Porcine serum has shown proliferation effects comparable to or exceeding those of FBS in satellite cells derived from calves and dairy cows. Moreover, complete replacement of FBS with porcine serum in cultures of human adipose-derived mesenchymal stem cells and porcine skeletal muscle satellite cells maintained cell viability, proliferation, and tissue-specific differentiation (Hahn et al., 2024; Tavsanli et al., 2025). Moreover, black-boned chicken (BBC) serum has been reported to outperform FBS in the culture of BBC embryonic stem cells (Promtan et al., 2023). Lee et al. (2023, 2024b) compared the culture efficiency of bovine, porcine, and chicken sera in bovine muscle satellite cells and found that bovine and porcine sera supported cell viability comparable to FBS, while bovine and chicken sera promoted 70–75% greater muscle formation.

Despite these advances, research on using visceral by-products in place of FBS remains nonexistent. This lack of research may be explained by the fact that visceral tissues have traditionally been consumed as food or discarded due to hygiene concerns and cultural perceptions, and they have often been regarded as peripheral or low-priority resources. By contrast, livestock blood has attracted comparatively greater research attention as a candidate material, largely because FBS is blood-derived and consequently perceived as a more viable alternative. Overall, slaughterhouse blood demonstrates considerable potential as a serum substitute; nevertheless, comprehensive research on the broader utilization of slaughterhouse by-products is required to enhance their value and to foster the development of sustainable and effective alternatives to FBS.

## Conclusion

This study reviewed the utilization of by-products obtained during the slaughtering of major livestock species for food and biomaterial applications. It also discussed the definition and role of FBS as a key supplement in cell culture, research trends focused on its replacement, and the potential applications of slaughterhouse by-products. Livestock by-products have been traditionally used as food ingredients in Asia, Europe, and the Americas and have also found applications as additives in diverse food products, pharmaceuticals, and biomaterials. However, their use as food materials is typically limited to traditional preparations or incorporation as secondary ingredients, with limited exploration of novel applications.

The review highlighted that while the composition of blood components is broadly similar across livestock species, the specific content of individual components varies depending on factors such as species, breed, sex, rearing period, and feed. These variations can complicate direct comparisons; nevertheless, the similarities suggest that such by-products may be repurposed for similar uses. Notably, several studies have investigated blood-derived products, demonstrating the potential of bovine, porcine, and chicken sera as FBS alternatives. In contrast, research on the use of other by-products, such as viscera, remains limited.

Despite the large volume of slaughter by-products produced, their utilization in cell culture remains underutilized. Therefore, strategic research is urgently needed to enhance the added value of these by-products and to develop functional materials that support sustainable cultured meat production. In parallel, the development of immortalized cell lines to ensure a stable cell supply and the optimization of culture conditions are crucial steps toward improving the economic feasibility and safety of cultured meat production.

In conclusion, replacing FBS and utilizing slaughter by-products are expected to play critical roles in the sustainable development of the cultured meat industry and the establishment of scalable production systems. Future research should focus on the in-depth characterization of various by-products, the development of customized culture media to efficiently utilize these resources, and the holistic improvement of production systems, with attention to ethical and environmental considerations. These efforts will support the commercialization of cultured meat while addressing global concerns regarding animal welfare and environmental sustainability.

## Supplementary Information

Below is the link to the electronic supplementary material.

Supplementary Material 1

## Data Availability

No datasets were generated or analysed during the current study.
