# Supplementary material for: Utilization of animal by-products as sources of bioactive compounds and FBS alternatives for cultured meat: a comprehensive review
Source: Food Sci Anim Resour. 2026 Feb 11;46(1):32. doi: 10.1007/s44463-025-00035-8 (PMC12995063; doi:10.1007/s44463-025-00035-8)
Supplement: Supplementary file 1 — Supplementary Material 1 [file 44463_2025_35_MOESM1_ESM.docx]

**Supplementary information**

**Journal: Food science of animal resources**

**Development of the various food materials and fetal bovine serum substitute for cultured meat using animal by-products**

**References for Table 1**

Bulk supplements. Beef born broth protein powder. 2024. https://www.bulksupplements.com/ko/products/%EC%87%A0%EA%B3%A0%EA%B8%B0-%EB%BC%88-%EA%B5%AD%EB%AC%BC-%EB%8B%A8%EB%B0%B1%EC%A7%88. Accessed April 23 2025.

Freja Foods. Beef Bone Broth. 2025. https://frejafoods.com/products/beef-bone-broth. Accessed April 24 2025.

US Wellness Meats. Beef Head Cheese. 2022. https://grasslandbeef.com/products/grassfed-beef-head-cheese?_pos=1&_sid=b4454f55c&_ss=r. Accessed April 23 2025.

Lineage Provisions. 100% Grass-Fed Beef & Organ Meat Sticks. 2025. https://lineageprovisions.com/products/classic-meat-sticks. Accessed April 24 2025.

Mehmood L, Mujahid SA, Asghar S, ur Rahman HU, Khalid N. Formulation and quality evaluation of chicken nuggets supplemented with beef and chicken livers. Food Sci Anim Resour. 2024; https://doi.org/10.5851/kosfa.2024.e7

Carnivore Crisps. Carnivore Crumbs 1.5 oz liver. 2025. https://carnivorecrisps.com/products/carnivore-crumbs-1-5-oz-liver. Accessed April 23 2025.

Juanita's Foods. Menudo with honeycomb tripe. 2024. https://www.juanitas.com/products/menudo/honeycomb-tripe/. Accessed April 24 2025.

Marks and Spencer. Cured Ox Tongue. 2025. https://www.marksandspencer.com/food/cured-ox-tongue/p/fdp60004677. Accessed April 24 2025.

Paulina Market. Jellied Calves Tongue. 2025. htps://www.paulinamarket.com/products/jellied-calves-ongue?pr_prod_strat=jac&pr_rec_id=4d805a5ac&pr_rec_pid=913416195&pr_ref_pid=916095363&pr_seq=uniform. Accessed April 24 2025.

Sohn JW, Yum CA, Kim CJ. Effects of binding materials on sensory and cooking properties of Soondae. Korean J Food Cook Sci. 1999;(2):191–196.

Anjos O, Fernandes R, Cardoso SM, Delgado T, Farinha N, Paula V, Carpes ST. Bee pollen as a natural antioxidant source to prevent lipid oxidation in black pudding. Lwt. 2019; https://doi.org/10.1016/j.lwt.2019.05.105

Mama Tong Soup. Pork Bone Broth. 2025. https://mamatongsoup.com/products/pork-bone-broth-32oz-local-pickup. Accessed April 24 2025.

Choi YS, Ku SK, Lee HJ, Park JD, Sung JM, Jeon KH, Kim YB. Effects of pork liver levels on the quality characteristics on hamburger patties. Korean J Food Cook Sci. 2017; https://doi.org/10.9724/kfcs.2017.33.1.20

Essentia Protein Solution. Pork liver pate. 2025. https://essentiaproteins.com/int/applications/inspiration/meat-products/emulsified/000257-pork-liver-pate-with-t-92-sf/. Accessed April 23 2025.

4505 meats. Chicharrones. 2025. https://4505meats.com/. Accessed July 15 2025.

Sriwattana S, Utama-ang N, Thakeow P, Senapa J, Phimolsiripol Y, Surawang S, Angeli S. Physical, chemical and sensory characterization of the Thai-crispy pork rind ‘Kaeb Moo’. Chiang Mai Univ J Nat Sci Spec Issue Agric Nat Resour 2012;11(1):181–191.

Foodmax. Charcoal grilled boneless chicken feet. 2020. https://foodmax.co.kr/shop/item.php?it_id=1550852306. Accessed April 23 2025.

Edenfarmer. Pattes de Poulet congelées. 2023. https://edenfarmer.com/produit/pattes-de-poulet-congelees/. Accessed April 23 2025.

Sanderson Farms. Generic chicken gizzards. 2025. https://sandersonfarms.com/products/gizzards/. Accessed July 15 2025.

Lifedryfish. Fried chicken gizzard. 2024. https://lifedryfish.com/65/?idx=283. Accessed April 23 2025.

Foodjang. Grilled salted skewer. 2017. https://www.foodjang.com/goods/goods_view.php?goodsNo=1000000052#detail. Accessed April 23 2025.

TC.FARM. Chicken Hearts. 2023. https://tc.farm/chicken-hearts/. Accessed April 23 2025.

Ono T, Salat H. The Japanese grill: from classic yakitori to steak, seafood, and vegetables [a cookbook]. Berkeley (CA): Ten Speed Press; 2011.

Citarella. Citarella Chopped Chicken Liver Spread. 2015. https://shoplocal.citarella.com/store/citarella/products/25431135-chopped-chicken-liver-spread-8-oz. Accessed April 23 2025.

Carrefour. Foie de volaille confits CARREFOUR EXTRA. 2022. https://www.carrefour.fr/p/foie-de-volaille-confits-carrefour-extra-3270190265436. Accessed April 23 2025.

Schallerweber. Chicken Liver Pâté. 2025. https://schallerweber.com/products/chicken-liver-pate?_pos=1&_sid=653c8222f&_ss=r. Accessed July 15 2025.

Dewoeste grond. Organic chicken liver sausage. 2025. https://www.dewoestegrond.nl/en/bio-chicken-liver-sausage.html. Accessed April 23 2025.

Chickencrackling. Oursnack. 2022. https://www.chickencrackling.com/#oursnacks. Accessed April 23 2025.

Jinji. Judang Restaurant Fried Chicken Skin. 2025. https://m.jinjimarket.com/. Accessed July 15 2025.

Togetherfarms. Lamb Heart. 2024. https://togetherfarms.com/store/product/organs-heart-lamb. Accessed April 23 2025.

Sainsburys. Sainsbury's British or New Zealand Lamb Liver. 2018. https://www.sainsburys.co.uk/gol-ui/product/sainsburys-fresh-lambs-liver-approx-450g-. Accessed April 23 2025.

Unclebeef. Skilpadjies. 2021. https://unclebeef.co.uk/product/skilpadjies-lambs-liver/. Accessed April 23 2025.

Lanaucelloise. Jar of Charles SAVY lamb tripous. 2015. https://www.lanaucelloise.fr/boutique/en/the-tripous/charles-savy-naucellois-tripou/jar-of-4-charles-savy-lamb-tripous147-_BQ.php. Accessed April 23 2025.

Finefoodspecialist. Lamb’s Tongue Tacos. 2019. https://www.finefoodspecialist.co.uk/drogos-kitchen/lamb-s-tongue-tacos. Accessed April 23 2025.

Kimmart. DUCK FEET. 2025. https://sskim.com.sg/duck-feet-1kg-pkt-sg. Accessed April 23 2025.

Bella Bella Gourmet. Duck confit gizzards. 2025. https://bellabellagourmet.com/products/duck-gizzard-confit?srsltid=AfmBOoqwa5lLIndzfBJxvDR6wJpphl43Fy3ggOkWfkG2r4Q-uO0XzY4f. Accessed July 16 2025.

Reatonfood. Duck liver. 2025. https://e-food.reatonfood.eu/en/3oi-129054204. Accessed April 23 2025.

Kendo. Duck Liver. 2021. https://kendo.com.sg/fresh-meat/duck/duck-liver. Accessed April 23 2025.

XIAO HU DUCK. Sweet & Spicy Duck Tongue. 2025. https://en.xiaohuya.com.cn/proDetail/13.html. Accessed July 15 2025.

**References for Table 2**

Boles JA, Rathgeber BM, Shand PJ. Recovery of proteins from beef bone and the functionality of these proteins in sausage batters. Meat Sci. 2000; https://doi.org/10.1016/S0309-1740(99)00146-1

Ahmad T, Ismail A, Ahmad SA, Khalil KA, Teik Kee L, Awad EA, Sazili AQ. Physicochemical characteristics and molecular structures of gelatin extracted from bovine skin: effects of actinidin and papain enzymes pretreatment. Int J Food Prop. 2019; https://doi.org/10.1080/10942912.2019.1576731

Said MI, Heryanto H, Tahir D. Hydroxyapatite (HA) synthesis from leg bone by-product of beef cattle: structural and optical characteristics for various sintering temperatures. JOM. 2024; https://doi.org/10.1007/s11837-024-06431-7

Song Z, Ni W, Li B, Ma Y, Han L, Yu Q. Sustainable ferritin from bovine by-product liver as a potential resource: ultrasound assisted extraction and physicochemical, structural, functional, and stable analysis. Int J Biol Macromol. 2024; https://doi.org/10.1016/j.ijbiomac.2024.136264

Darine S, Christophe V, Gholamreza D. Production and functional properties of beef lung protein concentrates. Meat Sci. 2010; https://doi.org/10.1016/j.meatsci.2009.03.007

Noorzai S, Verbeek CJR, Lay MC, Swan J. Collagen extraction from various waste bovine hide sources. Waste Biomass Valorization. 2020; https://doi.org/10.1007/s12649-019-00843-2

Chang CY, Wu KC, Chiang SH. Antioxidant properties and protein compositions of porcine haemoglobin hydrolysates. Food Chem. 2007; https://doi.org/10.1016/j.foodchem.2005.12.019

Borges S, Piccirillo C, Scalera F, Martins R, Rosa A, Couto JA, et al. Valorization of porcine by-products: a combined process for protein hydrolysates and hydroxyapatite production. Bioresour Bioprocess. 2022; https://doi.org/10.1186/s40643-022-00522-6

Wakamatsu JI, Murakami N, Nishimura T. A comparative study of zinc protoporphyrin IX‐forming properties of animal by‐products as sources for improving the color of meat products. Anim Sci J. 2015; https://doi.org/10.1111/asj.12326

López-Pedrouso M, Lorenzo JM, Bou R, Vazquez JA, Valcarcel J, Toldrà M, Franco D. Valorisation of pork by-products to obtain antioxidant and antihypertensive peptides. Food Chem. 2023; https://doi.org/10.1016/j.foodchem.2023.136351

Gorlov IF, Titov EI, Semenov GV, Slozhenkina MI, Sokolov AY, Omarov RS, et al. Collagen from porcine skin: a method of extraction and structural properties. Int J Food Prop. 2018; https://doi.org/10.1080/10942912.2018.1466324

Toldrà M, Parés D, Saguer E, Carretero C. Recovery and extraction of technofunctional proteins from porcine spleen using response surface methodology. Food Bioprocess Technol. 2019; https://doi.org/10.1007/s11947-018-2208-0

Lee SY, Kang HJ, Park Y, Hur SJ. Development of effective heparin extraction method from pig by-products and analysis of their bioavailability. J Anim Sci Technol. 2020; https://doi.org/10.5187/jast.2020.62.6.933

Damgaard TD, Otte JA, Meinert L, Jensen K, Lametsch R. Antioxidant capacity of hydrolyzed porcine tissues. Food Sci Nutr. 2014; https://doi.org/10.1002/fsn3.106

Wongngam W, Mitani T, Katayama S, Nakamura S, Yongsawatdigul J. Production and characterization of chicken blood hydrolysate with antihypertensive properties. Poult Sci. 2020; https://doi.org/10.1016/j.psj.2020.07.006

Rosa CS, Hoelzel SC, Viera VB, Barreto PM, Beirão LH. Atividade antioxidante do ácido hialurônico extraído da crista de frango. Cienc Rural. 2008; https://doi.org/10.1590/S0103-84782008005000031

Adler SA, Slizyte R, Honkapää K, Løes AK. In vitro pepsin digestibility and amino acid composition in soluble and residual fractions of hydrolyzed chicken feathers. Poult Sci. 2018; https://doi.org/10.3382/ps/pey175

Oluba OM, Obi CF, Akpor OB, Ojeaburu SI, Ogunrotimi FD, Adediran AA, Oki M. Fabrication and characterization of keratin starch biocomposite film from chicken feather waste and ginger starch. Sci Rep. 2021; https://doi.org/10.1038/s41598-021-88002-3

Matinong AME, Chisti Y, Pickering KL, Haverkamp RG. Collagen extraction from animal skin. Biology. 2022; https://doi.org/10.3390/biology11060905

Lazarini M, Bordeaux-Rego P, Giardini-Rosa R, Duarte AS, Baratti MO, Zorzi AR, de Miranda JB, Cesar CL, Luzo A, Saad STO. Natural type II collagen hydrogel, fibrin sealant, and adipose-derived stem cells as a promising combination for articular cartilage repair. Cartilage. 2017; https://doi.org/10.1177/1947603516675914

Stiborova H, Kronusova O, Kastanek P, Brazdova L, Lovecka P, Jiru M, Belkova B, Poustka J, Stranska M, Hajslova J, Demnerova K. Waste products from the poultry industry: a source of high‐value dietary supplements. J Chem Technol Biotechnol. 2020; https://doi.org/10.1002/jctb.6131

dos Santos Aguilar JG, de Souza AKS, de Castro RJS. Enzymatic hydrolysis of chicken viscera to obtain added-value protein hydrolysates with antioxidant and antihypertensive properties. Int J Pept Res Ther. 2020; https://doi.org/10.1007/s10989-019-09879-3

Saenmuang S, Phothiset S, Chumnanka C. Extraction and characterization of gelatin from black-bone chicken by-products. Food Sci Biotechnol. 2020; https://doi.org/10.1007/s10068-019-00696-4

Santra L, Gupta S, Kannan S, Singh AK, Ravi Kumar G, Naskar S, Ghosh J, Dhara SK. Long bones, a slaughterhouse by-product, may serve as an excellent source for mesenchymal stem cells. Indian J Anim Sci. 2017; https://doi.org/10.56093/ijans.v87i1.66860

Gavinho SR, Bozdag M, Kalkandelen C, Regadas JS, Jakka SK, Gunduz O, Oktar FN, Graça MPF. An eco-friendly process to extract hydroxyapatite from sheep bones for regenerative medicine: structural, morphologic and electrical studies. J Funct Biomater. 2023; https://doi.org/10.3390/jfb14050279

Kumar SL, Anandhavelu S, Sivaraman J, Swathy M. Modified extraction and characterization of keratin from Indian goat hoof: a biocompatible biomaterial for tissue regenerative applications. Integr Ferroelectr. 2017; https://doi.org/10.1080/10584587.2017.1368642

Singh H, Purohit SD, Bhaskar R, Yadav I, Gupta MK, Mishra NC. Development of decellularization protocol for caprine small intestine submucosa as a biomaterial. Biomater Biosyst. 2022; https://doi.org/10.1016/j.bbiosy.2021.100035

Balakrishnan B, Prasad B, Rai AK, Velappan SP, Subbanna MN, Narayan B. In vitro antioxidant and antibacterial properties of hydrolysed proteins of delimed tannery fleshings: comparison of acid hydrolysis and fermentation methods. Biodegradation. 2011; https://doi.org/10.1007/s10532-010-9398-0

Banerjee I, Mishra D, Das T, Maiti S, Maiti TK. Caprine (goat) collagen: a potential biomaterial for skin tissue engineering. J Biomater Sci Polym Ed. 2012; https://doi.org/10.1163/092050610X551943

de Queiroz ALM, de Araújo ARR, Pacheco MTB, Madruga MS. Potential use of goat viscera to obtain protein hydrolysates. LWT. 2017; https://doi.org/10.1016/j.lwt.2017.06.049

Hu X, Feng N, Zhang J. Study on the factors influencing the extraction of chenodeoxycholic acid from duck bile paste by calcium salt method. J Appl Chem. 2018; https://doi.org/10.1155/2018/7253639

Zheng Z, Wei X, Shang T, Huang Y, Hu C, Zhang R. Bioconversion of duck blood cell: process optimization of hydrolytic conditions and peptide hydrolysate characterization. BMC Biotechnol. 2018; https://doi.org/10.1186/s12896-018-0

**References for Table 3**

Alberghina D, Giannetto C, Vazzana I, Ferrantelli V, Piccione G. Reference intervals for total protein concentration, serum protein fractions, and albumin/globulin ratios in clinically healthy dairy cows. J Vet Diagn Invest. 2011; https://doi.org/10.1177/104063871102300119

Bah CS, Bekhit AEDA, Carne A, McConnell MA. Composition and biological activities of slaughterhouse blood from red deer, sheep, pig and cattle. J Sci Food Agric. 2016; https://doi.org/10.1002/jsfa.7062

Barrett SRW, Seymour RS. Extreme hypoxia and high lactate concentrations in early chicken embryos show that cutaneous oxygen uptake is limited by diffusion and metabolism is partially anaerobic. J Comp Physiol B. 2021; https://doi.org/10.1007/s00360-021-01372-y

Baudouin KA, Soualio KAMAGATE, Mathieu BN, Angoue YAPO. Hematological profile of broilers and local chickens in Korhogo, Cote d’Ivoire. Int J Agric Environ Bioresearc. 2021; https://doi.org/10.35410/IJAEB.2021.5618

Bigland CH, Triantaphyllopoulos DC. Chicken prothrombin, thrombin, and fibrinogen. Am J Physiol. 1961; https://doi.org/10.1152/ajplegacy.1961.200.5.1013

Clauser JC, Maas J, Mager I, Halfwerk FR, Arens J. The porcine abattoir blood model—evaluation of platelet function for in‐vitro hemocompatibility investigations. Artif Organs. 2022; https://doi.org/10.1111/aor.14146

Coghe J, Uystepruyst CH, Bureau F, Detilleux J, Art T, Lekeux P. Validation and prognostic value of plasma lactate measurement in bovine respiratory disease. Vet J. 2000; https://doi.org/10.1053/tvjl.2000.0487

Das GB, Ahad A, Hossain ME, Akbar MA, Akther S, Mahmood A. Effect of different oil supplements on humoral immune response and lipid profile in commercial broiler. Pakistan Vet J. 2014; 34(2):229–233.

EFSA Panel on Food Contact Materials, Enzymes, Flavourings and Processing Aids (CEF). Scientific Opinion on thrombin from cattle (bovines) and pig's blood. EFSA J. 2015; https://doi.org/10.2903/j.efsa.2015.4018

Elsayed M, Elkomy A, Aboubakr M, Morad M. Tissue residues, hematological and biochemical effects of tilmicosin in broiler chicken. Vet Med Int 2014; https://doi.org/10.1155/2014/502872

Esonu CE, Onyeze G, Iheanacho KM, Nwaogu LN, Odirichukwu SP. Extraction, purification and kinetic study of lactate dehydrogenase of male chicken from Ebocha-oil exploration area, Nigeria. Extraction. 2019; https://doi.org/10.9734/ajbgmb/2019/v2i430065

Filipovic N, Stojevic Z, Milinkovic-Tur S, Ljubic BB, Zdelar-Tuk M. Changes in concentration and fractions of blood serum proteins of chickens during fattening. Veterinarski Arhiv. 2007; 77(4):319.

Garcia RA, Nieman CM, Haylock RA, Rosentrater KA, Piazza GJ. The effect of chicken blood and its components on wastewater characteristics and sewage surcharges. Poult Sci 2016; https://doi.org/10.3382/ps/pew114

Gorlov IF, Levakhin VI, Radchikov VF, Tsai VP, Bozhkova SE. Effect of feeding with organic microelement complex on blood composition and beef production of young cattle. Mod Appl Sci. 2015; https://doi.org/10.5539/mas.v9n10p8

Hong D, Liyun C, Fuwei LI, Qiaoxian Y, Dehe W, Rongyan Z, Hui C. Research note: effect of age on hematological parameter and reference intervals for commercial Lohmann silver layer. Poult Sci. 2021; https://doi.org/10.1016/j.psj.2021.101497

Lange-Consiglio A, Garlappi R, Spelta C, Idda A, Comazzi S, Rizzi R, Cremonesi F. Physiological parameters to identify suitable blood donor cows for preparation of platelet rich plasma. Animals. 2021; http://doi.org/10.3390/ani11082296

Lee DY, Lee SY, Yun SH, Choi Y, Han D, Park J, Kim JS, Mariano E Jr, Lee J, Choi JS, Kim GD, Choi I, Joo ST, Hur SJ. Study on the feasibility of using livestock blood as a fetal bovine serum substitute for cultured meat. J Food Sci 2024; https://doi.org/10.1111/1750-3841.17347

Lee SY, Yun SH, Jeong JW, Kim JH, Kim HW, Choi JS, Kim G, Joo ST, Hur SJ. Review of the current research on fetal bovine serum and the development of cultured meat. Food Sci Anim Resour. 2022; https://doi.org/10.5851/kosfa.2022.e46

Lumsden JH, Mullen K, Rowe R. Hematology and biochemistry reference values for female Holstein cattle. Can J Comp Med. 1980; 44(1):24.

Mahoney CP, Alster FA, Carew Jr LB. Growth, thyroid function, and serum macromineral levels in magnesium-deficient chicks. Poult Sci. 1992; https://doi.org/10.3382/ps.0711669

Manunza A, Casellas J, Quintanilla R, González-Prendes R, Pena RN, Tibau J, Mercadé A, Castelló A, Aznárez N, Hernández-Sánchez J, Amills M. A genome-wide association analysis for porcine serum lipid traits reveals the existence of age-specific genetic determinants. BMC Genomics. 2014; https://doi.org/10.1186/1471-2164-15-758

Mazur NP, Fedorovych VV, Fedorovych EI, Fedorovych OV, Bodnar PV, Gutyj BV, Kuziv MI, Kuziv NM, Orikhivskyi TV, Grabovska OS, Denys HH, Stakhiv NP, Hudyma VY, Pakholkiv NI. Effect of morphological and biochemical blood composition on milk yield in Simmental breed cows of different production types. Ukr J Ecol. 2020; https://doi.org/doi: 10.15421/2020_110

Mosher DF, Blout ER. Heterogeneity of bovine fibrinogen and fibrin. J Biol Chem. 1973; https://doi.org/10.1016/S0021-9258(19)43434-0

Nwaigwe CU, Ihedioha JI, Shoyinka SV, Nwaigwe CO. Evaluation of the hematological and clinical biochemical markers of stress in broiler chickens. Vet World. 2020; https://doi.org/10.14202/vetworld.2020.2294-2300

Osadcha Y, Shuliar A, Sydorenko O, Dzhus P, Shuliar A. Biochemical parameters of chicken blood under the influence of technological stimuli of various etiologies. Sci Horizons. 2023; https://doi.org/10.48077/scihor9.2023.70

Pampori ZA, Iqbal S. Haematology, serum chemistry and electrocardiographic evaluation in native chicken of Kashmir. Int J Poult Sci. 2007; https://doi.org/10.3923/ijps.2007.578.582

Rowlands GJ, Stark AJ, Manston R, Lewis WH, Saunders RW. The blood composition of different breeds of bulls undergoing beef performance tests. Res Vet Sci. 1977; https://doi.org/10.1017/S0022029900019889

Schaefer AL, Doornenbal H, Sather AP, Tong AKW, Jones SDM, Murray AC. The use of blood serum components in the identification of stress-susceptible and carrier pigs. Can J Anim Sci. 1990; https://doi.org/10.4141/cjas90-103

Sobiech P, Kuleta Z, Jalynski M. Serum LDH isoenzyme activity in dairy and beef cows. Acta Sci Pol Med Vet. 2002; 1:39–43.

Sorapukdee S, Narunatsopanon S. Comparative study on compositions and functional properties of porcine, chicken and duck blood. Korean J Food Sci Anim Resour. 2017; https://doi.org/10.5851/kosfa.2017.37.2.228

St. Paul M, Paolucci S, Barjesteh N, Wood RD, Schat KA, Sharif S. Characterization of chicken thrombocyte responses to Toll-like receptor ligands. PLOS ONE. 2012; https://doi.org/10.1371/journal.pone.0043381

Xuan NH, Loc HT, Ngu NT. Blood biochemical profiles of Brahman crossbred cattle supplemented with different protein and energy sources. Vet World. 2018; https://doi.org/10.14202/vetworld.2018.1021-1024

Yadav SP, Kannaki TR, Mahapatra RK, Paswan C, Bhattacharya TK, Sarkar SK, Chatterjee RN. In vivo cell-mediated immune, hemagglutination inhibition response, hematological and biochemical values in native vs. exotic chicken breeds. Poult Sci 2018; https://doi.org/10.3382/ps/pey182

Zaitsev SY, Belous AA, Voronina OA, Rykov RA, Bogolyubova NV. Correlations between antioxidant and biochemical parameters of blood serum of Duroc breed pigs. Animals 2021; https://doi.org/10.3390/ani11082400

Żbikowski A, Pawłowski K, Śliżewska K, Dolka B, Nerc J, Szeleszczuk P. Comparative effects of using new multi-strain synbiotics on chicken growth performance, hematology, serum biochemistry and immunity. Animals. 2020; https://doi.org/10.3390/ani10091555
